# Transcriptomic comparison between developing seeds of yellow- and black-seeded *Brassica napus* reveals that genes influence seed quality

**DOI:** 10.1186/s12870-019-1821-z

**Published:** 2019-05-16

**Authors:** Jinjin Jiang, Shuang Zhu, Yi Yuan, Yue Wang, Lei Zeng, Jacqueline Batley, You-Ping Wang

**Affiliations:** 1 Jiangsu Provincial Key Laboratory of Crop Genetics and Physiology, Yangzhou University, Yangzhou, 225009 China; 2 School of Biological Sciences, University of Western Australia, Perth, WA Australia

**Keywords:** *Brassica napus*, Seed coat color, Flavonoid biosynthesis, Fatty acid, Gene expression

## Abstract

**Background:**

*Brassica napus* is of substantial economic value for vegetable oil, biofuel, and animal fodder production. The breeding of yellow-seeded *B. napus* to improve seed quality with higher oil content, improved oil and meal quality with fewer antinutrients merits attention. Screening the genes related to this phenotype is valuable for future rapeseed breeding.

**Results:**

A total of 85,407 genes, including 4317 novel genes, were identified in the developing seeds of yellow- and black-seeded *B. napus*, and yellow rapeseed was shown to be an introgression line between black-seeded *B. napus* and yellow-seeded *Sinapis alba*. A total of 15,251 differentially expressed genes (DEGs) were identified among all the libraries, and 563 and 397 common DEGs were identified throughout black and yellow seed development, including 80 upregulated and 151 downregulated genes related to seed development and fatty acid accumulation. In addition, 11 up-DEGs and 31 down-DEGs were identified in all developmental stages of yellow rapeseed compared with black seed. Enrichment analysis revealed that many DEGs were involved in biosynthetic processes, pigment metabolism, and oxidation-reduction processes, such as flavonoid and phenylpropanoid biosynthesis, phenylalanine metabolism, flavone and flavonol biosynthesis, and fatty acid biosynthesis and metabolism. We found that more than 77 DEGs were related to flavonoid and lignin biosynthesis, including *4CL*, *C4H*, and *PAL,* which participated in phenylalanine metabolism, and *BAN*, *CHI/TT5*, *DFR*, *F3H*, *FLS*, *LDOX*, *PAP*, *CHS/TT4*, *TT5*, *bHLH/TT8*, *WD40*, *MYB*, *TCP*, and *CYP*, which were involved in flavonoid biosynthesis. Most of these DEGs were downregulated in yellow rapeseed and were consistent with the decreased flavonoid and lignin contents. Both up- and down-DEGs related to fatty acid biosynthesis and metabolism were also analyzed, which could help to explain the improved oil content of yellow rapeseed.

**Conclusion:**

This research provided comprehensive transcriptome data for yellow-seeded *B. napus* with a unique genetic background, and all the DEGs in comparison with the black-seeded counterpart could help to explain seed quality differences, such as lower pigmentation and lignin contents, and higher oil content.

**Electronic supplementary material:**

The online version of this article (10.1186/s12870-019-1821-z) contains supplementary material, which is available to authorized users.

## Background

*Brassica napus,* as the third leading oilseed crop grown worldwide, greatly contributes to providing edible oils, biofuels, and animal fodder [1]. Rapeseed with increased oil content, better oil and meal quality, and improved yield has been the main breeding drive during the past decades [2]. Yellow-seeded *B. napus* was improved with better seed oil and meal quality due to fewer pigments and polyphenols; thus, breeding yellow rapeseed has been preferred for decades, and studies on the molecular mechanism of this phenotype have been reported as well [3]. Fewer antinutrients, including phenolic compounds, tannins, proanthocyanins (PAs), and lignin, in yellow rapeseed were correlated with the flavonoid biosynthetic, phenylpropanoid, and phenylalanine/tyrosine metabolic pathways with common substrates, such as coumaroyl CoA, caffeoyl CoA, and feruloyl CoA [4–6]. Hitherto, yellow-seeded *B. napus* has been selected from interspecific hybridization of *Brassica* species and intergeneric hybridization [7, 8]. As reported in *Arabidopsis thaliana*, mutations in *transparent testa* (*TT*) genes are responsible for seed coat color variations, including early (EBGs) and late (LBGs) biosynthesis genes [9]. The EBGs include *TT4*/*CHS*, *TT5*/*CHI*, *TT6*/*F3H*, and *TT7*/*F3’H*, and LBGs include *TT3*/*DFR*, *TT18*/*LDOX*/*ANS*, *BAN*/*ANR*, *TT12*, *TT19*/*GSTF12*/*GST26*, and *AHA10*. In addition, the MYB-bHLH-WD40 (MBW) complex has been reported to have important regulatory functions in anthocyanin accumulation. Four MBW have been reported, including TT2-TT8-TTG1, MYB5-TT8-TTG1, TT2-EGL3-TTG1, and TT2-GL3-TTG1 [10]. Appelhagen et al. (2011) found that PAP1/MYB75 and PAP2/MYB90 participated in the formation of MBW [11]. Homologous *TT*s related to seed coat variation in Brassicaceae have been delineated, including 95 copies of 21 *TTs* in *B. napus* [3]. Previously, *F3’H*, *TT2*, *PAL*, *BAN*, *TTG1*, *TT10*, and *TT1* were cloned and shown to have functions in flavonoid biosynthesis [12–19]. Yu (2013) reviewed the molecular mechanism of manupulating seed coat color in *Brassica* species, including the homologous *TTs* cloned in *Brassicas* [20]. Besides, molecular markers have also been developed for yellow-seeded *B. rapa* [21–23], *B. juncea* [24, 25] and *B. napus*. Liu et al. (2012) reported a lignin biosynthesis gene, *BnCCR1*, associated with yellow seed character, indicating a strong correlation between seed color and lignin content [26]. Stein et al. (2013) found that *BnAHA10* had effects on seed color and lignin content [27]. Wang et al. (2017) identified 22 single nucleotide polymorphisms (SNPs) on 7 chromosomes associated with seed coat color using a genome wide association study (GWAS) [28]. Functional analysis of *BnTT10* and *BnTT1* revealed that they are involved in PA metabolism, lignin synthesis, seed coat pigmentation, and fatty acid (FA) biosynthesis [18, 19]. However, due to the genome complexity of *B. napus* and the sensitivity of yellow seed color to environmental influences (e.g., light, temperature, fertilizers, and harvesting time), the molecular mechanisms of this phenotype remained unclear until now [29, 30].

The short domestication history of *B. napus* after interspecific hybridization between *B. rapa* and *B. oleracea* and the artificial selection during rapeseed breeding have greatly narrowed the genetic background [31]. No yellow-seed germplasm has been found in natural *B. napus*, and most of the yellow-seeded *B. napus* were created by crossing between *Brassica* species, such as hybrids between *B. napus* and *B. juncea*, *B. napus* and *B. carinata*, *B. juncea* and *B. oleracea* [32, 33]. Wild species in Brassicaceae, such as *Sinapis alba*, *S. arvensis*, *Camelina sativa*, *Crambe abyssinica*, and *Descurainia sophia*, exhibit many desirable characteristics, such as yellow seed coat, high erucic acid, pod shattering resistance, high unsaturated FA contents, and resistance to various diseases and abiotic stresses [34–36]. Introducing gene resources from these wild species would help to enrich the genetic background of *B. napus*, accompanying desirable traits. Zhang et al. (2009) obtained yellow-seeded rapeseed from intergeneric hybrids between *B. napus* and *D. sophia* [37]. Previously, Wang et al. (2005) created somatic hybrids between *B. napus* and *S. alba* [38] and selected yellow-seeded *B. napus* from hybrid progenies with improved oil content and decreased antinutrients [8, 39, 40]. In the present study, we used RNA-Seq to identify the expression differences at different seed developmental stages of yellow- and black-seeded *B. napus*, revealing the expression patterns of genes involved in various biological pathways related to seed coat pigmentation, FA biosynthesis and metabolism.

## Results

### Transcriptome sequencing and read mapping of yellow- and black-seeded *B. napus*

**Fig. 1:**
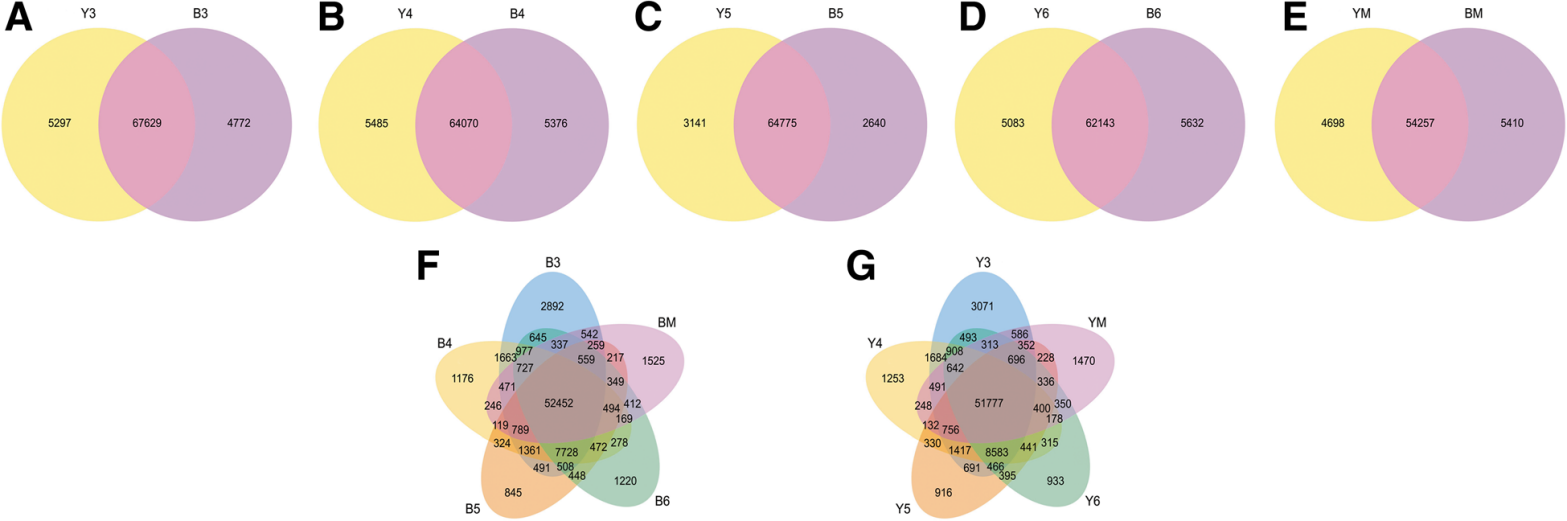
Genes commonly and specifically expressed in developing seeds of yellow- and black-seeded *B. napus*. **a**-**e** Venn diagram of genes expressed between each developmental stage of yellow and black seed. **f** Venn diagram of genes in black seed. **g** Venn diagram of genes in yellow seed. B3, B4, B5, B6 and BM indicate 3 ~ 6 WAF and mature black seed. Y3, Y4, Y5, Y6 and YM indicate 3 ~ 6 WAF and mature yellow seed

RNA-Seq was performed at three weeks after flowering (3 WAF), 4 WAF, 5 WAF, 6 WAF, and mature stage of yellow- and black-seeded *B. napus* to investigate the transcriptome difference that might be related to the quality variation between two rapeseeds. After quality control, ~ 30.3 to ~ 37.6 million reads from the libraries were uniquely mapped to the *B. napus* genome (Table 1). A total of 85,407 genes, including 4317 novel genes, were expressed in the developing seeds of two rapeseeds (Fig. 1, Additional file 1: Table S1). We found 52,452 and 51,777 common genes were expressed in the developing seeds of yellow and black rapeseed, respectively. In addition, 67,629 (3 WAF), 64,070 (4 WAF), 64,775 (5 WAF), 62,143 (6 WAF), and 54,257 (mature stage) overlapping genes were expressed at each stage in two rapeseed lines. Besides, 47,912 genes were coexpressed in all the developmental stages of yellow and black rapeseed lines.

**Table 1:** Summary of read mapping for RNA-seq

Sample name	B3	B4	B5	B6	BM	Y3	Y4	Y5	Y6	YM
Raw reads	47,536,866	42,753,820	44,341,556	51,507,518	51,272,946	48,704,282	45,694,038	44,797,562	45,996,584	52,804,244
Clean reads	45,555,018	41,801,216	40,620,518	43,833,034	48,665,348	46,813,976	43,798,322	42,843,336	43,736,106	50,268,482
Total mapped	36,499,205 (80.12%)	34,214,293 (81.85%)	32,844,328 (80.86%)	35,032,820 (79.92%)	39,325,179 (80.81%)	38,116,991 (81.42%)	35,893,586 (81.95%)	35,376,347 (82.57%)	36,029,106 (82.38%)	40,839,634 (81.24%)
Multiple mapped	2,175,877 (4.78%)	2,332,136 (5.58%)	2,561,972 (6.31%)	3,271,514 (7.46%)	3,151,365 (6.48%)	2,327,452 (4.97%)	2,287,911 (5.22%)	2,274,889 (5.31%)	2,451,932 (5.61%)	3,198,280 (6.36%)
Uniquely mapped	34,323,328 (75.34%)	31,882,157 (76.27%)	30,282,356 (74.55%)	31,761,306 (72.46%)	36,173,814 (74.33%)	35,789,539 (76.45%)	33,605,675 (76.73%)	33,101,458 (77.26%)	33,577,174 (76.77%)	37,641,354 (74.88%)
Reads map to ‘+’	17,137,064 (37.62%)	15,873,247 (37.97%)	15,138,033 (37.27%)	15,900,632 (36.28%)	18,123,297 (37.24%)	17,872,927 (38.18%)	16,769,787 (38.29%)	16,508,902 (38.53%)	16,764,771 (38.33%)	18,866,098 (37.53%)
Reads map to ‘-’	17,186,264 (37.73%)	16,008,910 (38.3%)	15,144,323 (37.28%)	15,860,674 (36.18%)	18,050,517 (37.09%)	17,916,612 (38.27%)	16,835,888 (38.44%)	16,592,556 (38.73%)	16,812,403 (38.44%)	18,775,256 (37.35%)
Non-splice reads	21,266,684 (46.68%)	21,368,313 (51.12%)	21,381,177 (52.64%)	22,323,651 (50.93%)	24,534,251 (50.41%)	22,491,873 (48.05%)	22,291,894 (50.9%)	24,114,006 (56.28%)	24,559,779 (56.15%)	26,061,989 (51.85%)
Splice reads	13,056,644 (28.66%)	10,513,844 (25.15%)	8,901,179 (21.91%)	9,437,655 (21.53%)	11,639,563 (23.92%)	13,297,666 (28.41%)	11,313,781 (25.83%)	8,987,452 (20.98%)	9,017,395 (20.62%)	11,579,365 (23.04%)

B3, B4, B5, B6 and BM indicate 3 ~ 6 WAF and mature black seed. Y3, Y4, Y5, Y6 and YM indicate 3 ~ 6 WAF and mature yellow seed

### Identification of differentially expressed genes

**Fig. 2:**
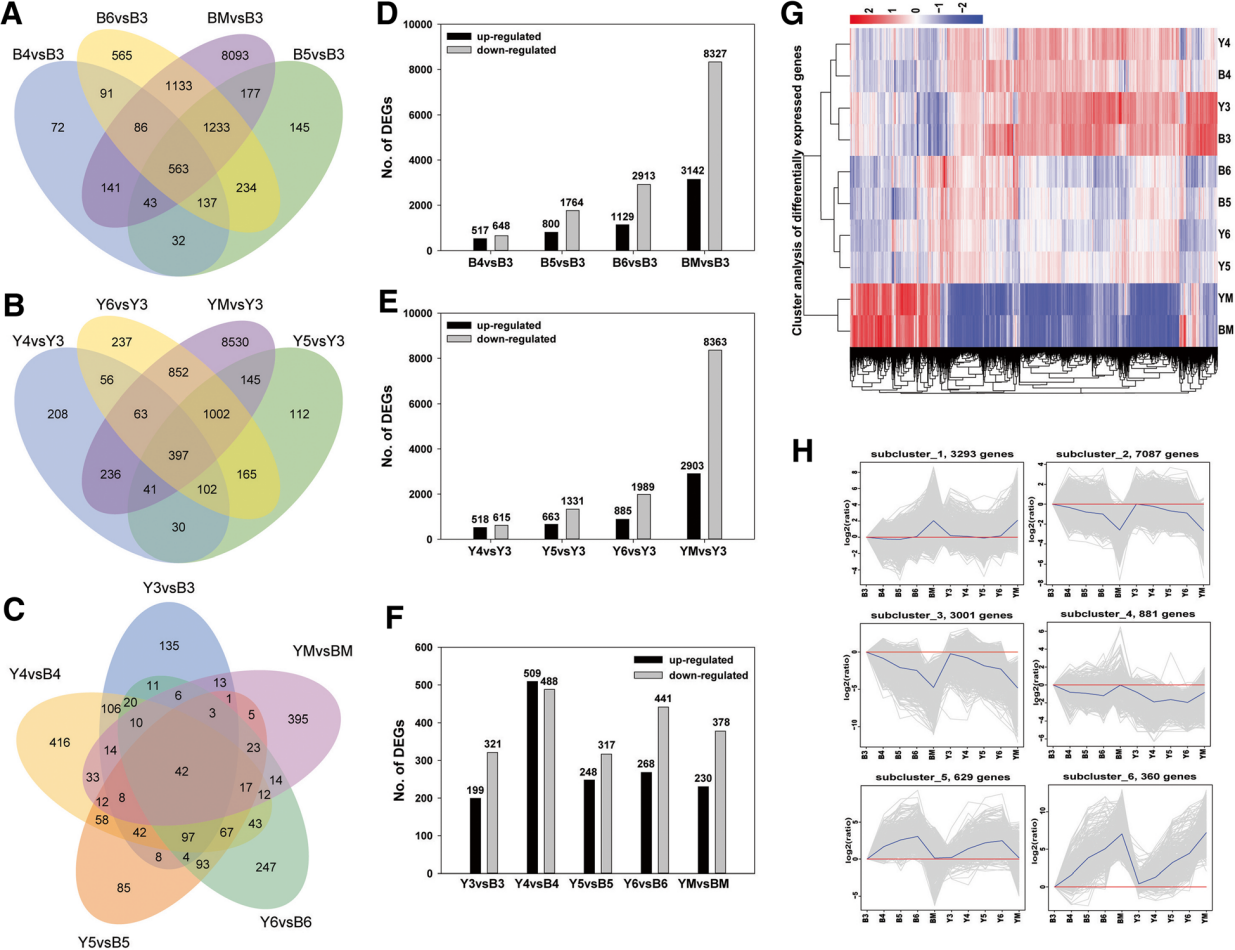
Expressional analysis of DEGs in developing seeds of yellow- and black-seeded *B. napus*. (**a**) DEGs with black seed development. (**b**) DEGs with yellow seed development. (**c**) DEGs between yellow and black rapeseed. (**d**-**f**) Number of up- and down-DEGs. (**g**-**h**) Clustering of DEGs

Global changes in differentially expressed genes were identified using DESeq, and a total of 15,251 DEGs (including 523 novel genes) were identified among all the libraries. We found 563 common DEGs in B4 (4 WAF of black seed), B5 (5 WAF of black seed), B6 (6 WAF of black seed), and BM (mature stage of black seed) compared with B3 (3 WAF of black seed), including 141 upregulated and 331 downregulated genes at each developmental stage of the black seed. A total of 397 common DEGs were identified in Y4 (4 WAF of yellow seed), Y5 (5 WAF of yellow seed), Y6 (6 WAF of yellow seed), and YM (mature stage of yellow seed) compared with Y3 (3 WAF of yellow seed), including 92 upregulated and 220 downregulated genes throughout yellow seed development (Fig. 2a, b; Additional file 2: Table S2). Generally, 80 upregulated and 151 downregulated genes were identified during the seed development of two *B. napus* lines, which might be related to seed development and FA accumulation (Additional file 3: Table S3). Those DEGs related to cruciferin, oleosin, caleosin, late embryogenesis abundant (LEA) hydroxyproline, lipid transfer protein, myrosinase-binding protein, embryo-specific protein, and alpha-tonoplast intrinsic protein were upregulated, and those related to galactosyltransferase, glycosyl hydrolase, lipid transfer protein, basic leucine zipper protein, cytochrome P450 (CYP), laccase, chalcone synthase, delta vacuolar processing enzyme, jasmonic acid carboxyl methyltransferase, and senescence were downregulated with seed development. In addition, 42 DEGs were identified between yellow and black rapeseeds at each stage, including 11 upregulated and 31 downregulated genes in yellow-seeded *B. napus* (Fig. 2c, Additional file 4: Table S4). These common DEGs were mainly related to the RNA-binding family protein, nascent polypeptide-associated complex (NAC), calcium binding EF-hand family protein, and metallothionein protein that were upregulated and dormancy/auxin associated protein, S-adenosylmethionine synthetase, pectin lyase-like superfamily protein, insulinase family protein that were downregulated in all stages of yellow seed compared with black seed. The number of DEGs increased with seed development in both rapeseed lines, whereas the downregulated DEGs at the mature stage dramatically increased to ~ 8000 (Fig. 2d, e). The up-regulated and downregulated DEGs between Y4 and B4 were higher than those in the other stages of the two rapeseed lines (Fig. 2f). Hierarchical cluster analysis of all the DEGs was performed using the log_10_(RPKM+ 1) value (Fig. 2g). H-cluster and SOM-cluster of the DEGs from five developmental stages of two rapeseed lines were also performed using the log_2_(Fold change), and the DEGs in all the clusters showed similar patterns between two rapeseed lines, except for subcluster_1_1, which includes DEGs with significant differences between Y4 and B4 (Fig. 2h, Additional file 5: Figure S1).

### Enrichment analysis of DEGs

**Fig. 3:**
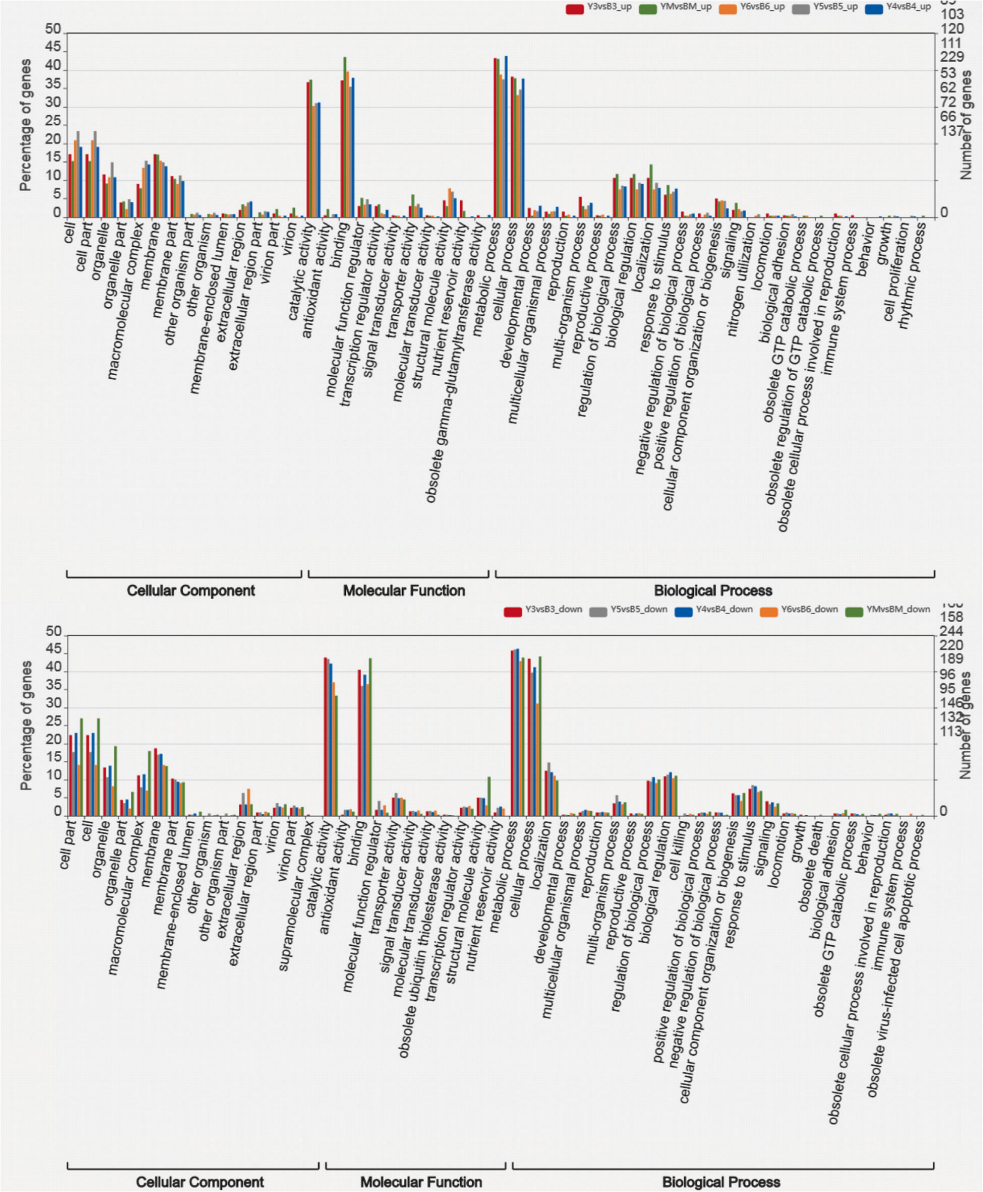
GO enrichment of up- and down-DEGs between yellow- and black-seeded *B. napus*

To acknowledge the putative functions and pathways of the DEGs, GO and KEGG enrichment analysis of DEGs was proceeded with GOseq and KOBAS 2.0. Among all the identified DEGs, 11,772 were annotated with GO terms and assigned to three categories: cellular component, biological process, and molecular function. Most of the up- and downregulated DEGs between the same stage of yellow and black seeds were assigned to catalytic activity, binding, metabolic process, cellular process, followed by cell, cell part, membrane, organelle part, macromolecular complex, regulation of biological process, and biological regulation (Fig. 3, Additional file 6: Table S5). In detail, among all the downregulated genes (1435 out of 1945 with GO annotations) between yellow and black rapeseed, 425, 39, 211, 89, and 65 DEGs were assigned to biosynthetic process (GO: 0009058), pigment metabolic process (GO: 0042440), oxidation-reduction process (GO: 0055114), cellular response to stimulus (GO: 0051716), and signal transduction (GO: 0007165), respectively. In contrast, 181 upregulated genes between yellow and black seed were assigned to biosynthetic process (GO: 0009058), 95 up-DEGs related to oxidation-reduction process (GO: 0055114), and 181 up-DEGs related to biosynthetic process (GO: 0009058).

KEGG pathway analysis showed that flavonoid biosynthesis was most significantly enriched with downregulated DEGs in comparisons of Y3 vs B3, Y4 vs B4, Y5 vs B5, and Y6 vs B6. Phenylpropanoid biosynthesis was also enriched with downregulated genes between the same stages of the two rapeseed lines, except for the mature stage. Phenylalanine metabolism, flavone and flavonol biosynthesis was enriched with downregulated DEGs in comparisons of Y3 vs B3 and Y4 vs B4 (Additional file 7: Figure S2). In contrast, the upregulated DEGs between yellow and black rapeseed were most significantly enriched in photosynthesis, followed by carbon metabolism, FA biosynthesis and metabolism (Additional file 8: Figure S3, Additional file 9: Table S6). The pathway enrichment analysis helps to determine the functions of DEGs and complemented secondary metabolisms that were not specified by GO terms. Using the log_2_(Fold change) values between yellow and black rapeseed (Y3 vs. B3, Y4 vs. B4, Y5 vs. B5, Y6 vs. B6, and YM vs. BM), we assigned all the DEGs to all the metabolism pathways using the *Arabidopsis* TAIR9 version as mapping reference. We also found many DEGs were enriched in metabolism (flavonoids, terpenes, phenylpropanoids and phenolics) and lipids (Additional file 10: Figure S4).

### DEGs associated with flavonoid and lignin biosynthesis

**Fig. 4:**
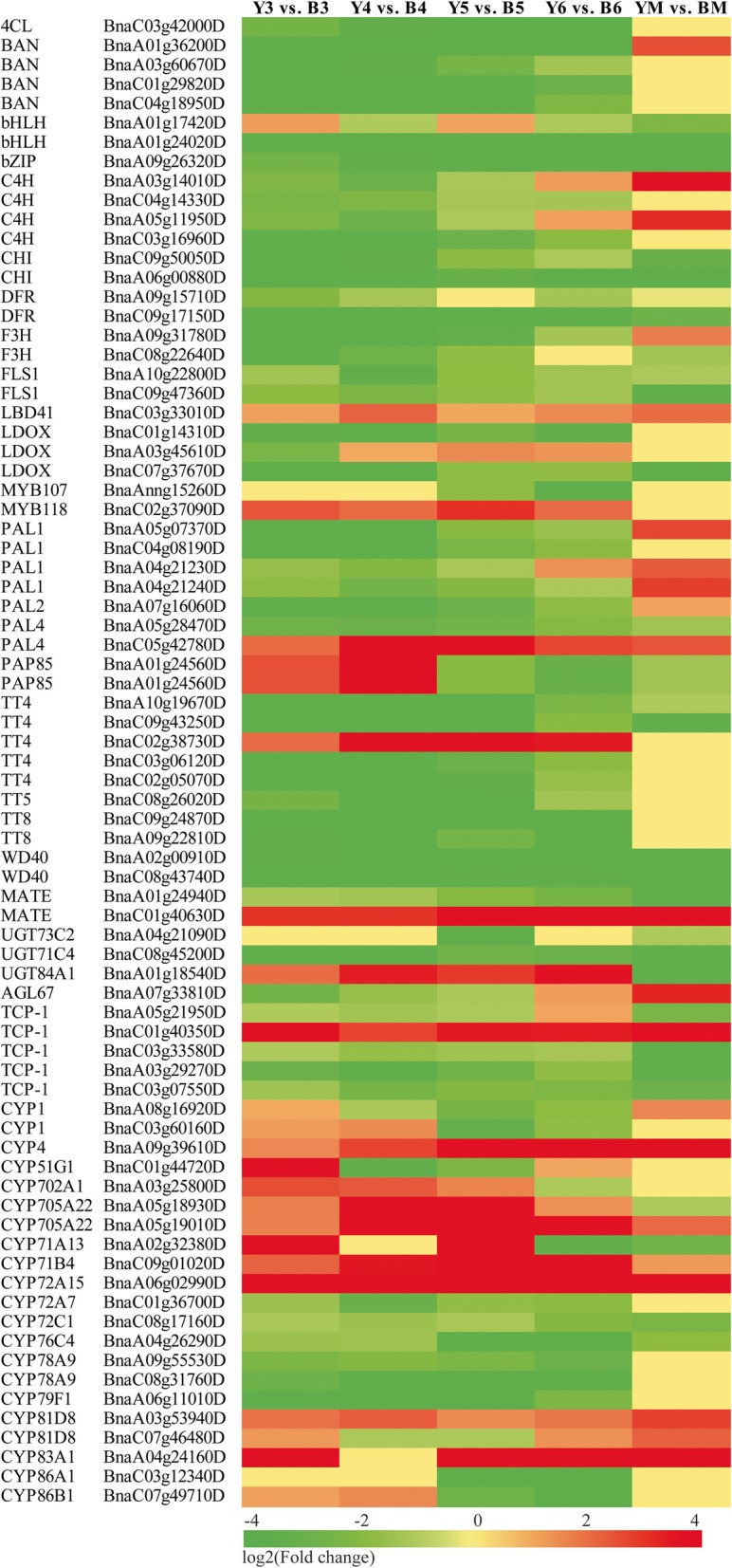
Heatmap of DEGs related to flavonoid and lignin biosynthesis

As mentioned above, yellow seed color is associated with flavonoid and lignin biosynthesis. We found 77 DEGs related to phenylpropanoid and flavonoid biosynthesis and the phenylalanine metabolic pathway. As shown in Fig. 4, genes encoding 4-coumarate CoA ligase (4CL), cinnamate 4-hydroxylase (C4H), and phenylalanine ammonia-lyase (PAL) were downregulated in yellow rapeseed compared with black seed, except for a homolog of *PAL* (BnaC05g42780D). These genes play important roles in lignin biosynthesis, and the expression changes should be related to the reduced lignin in yellow seed. Genes involved in the flavonoid pathway were also downregulated in yellow seed compared to black seed, including *BAN*, *CHI/TT5*, *DFR*, *F3H*, *FLS*, *LDOX*, *PAP*, *CHS/TT4*, *TT5*, *bHLH/TT8*, *WD40*, and *MYB*, which participate in the biosynthesis of chalcones, flavanones, flavonols, anthocyanins, and PAs. However, we found a *MATE* (BnaC01g40630D) and *UGT84A1* (BnaA01g18540D) were upregulated at the early developmental stages of yellow seed compared with black seed. Comparing with other two down-regulated MATEs in yellow-seeded *B. napus*, BnaA01g24940D (a homologous of AT3G03620) and BnaA07g18120D (a homologous of AtTT12), we found BnaC01g40630D with lower expression throughout rapeseed development than that of BnaA01g24940D and BnaA07g18120D. Expressional differences of *TCPs* and *CYPs* were also identified, which might be related to flavonoid biosynthesis, since TCP could interact with R2R3-MYB [41], and CYPs (e.g., CYP71/75/93/81) have been reported to function in the biosynthesis of anthocyanin pigments and condensed tannins [42]. In addition, we compared the expression pattern of all the genes involved in secondary metabolism and found DEGs encoding peroxidase superfamily protein (BnaA02g02010D, BnaC06g32660D, BnaA02g14050D, and BnaC06g21080D), glycosyl hydrolase family protein (BnaUnng02770D), beta glucosidase 19/29/25 (BnaA06g09700D, BnaA05g04040D, BnaC05g11190D, BnaC01g43700D, and BnaC01g40610D), thioglucoside glucohydrolase 1 (BnaCnng53320D) were also assigned to phenylpropanoid biosynthesis and the phenylalanine metabolic pathway (Additional file 11: Figure S5). Generally, most of the genes involved in secondary metabolism were downregulated in yellow seed compared with black seed.

### DEGs associated with fatty acid biosynthesis and metabolism

**Fig. 5:**
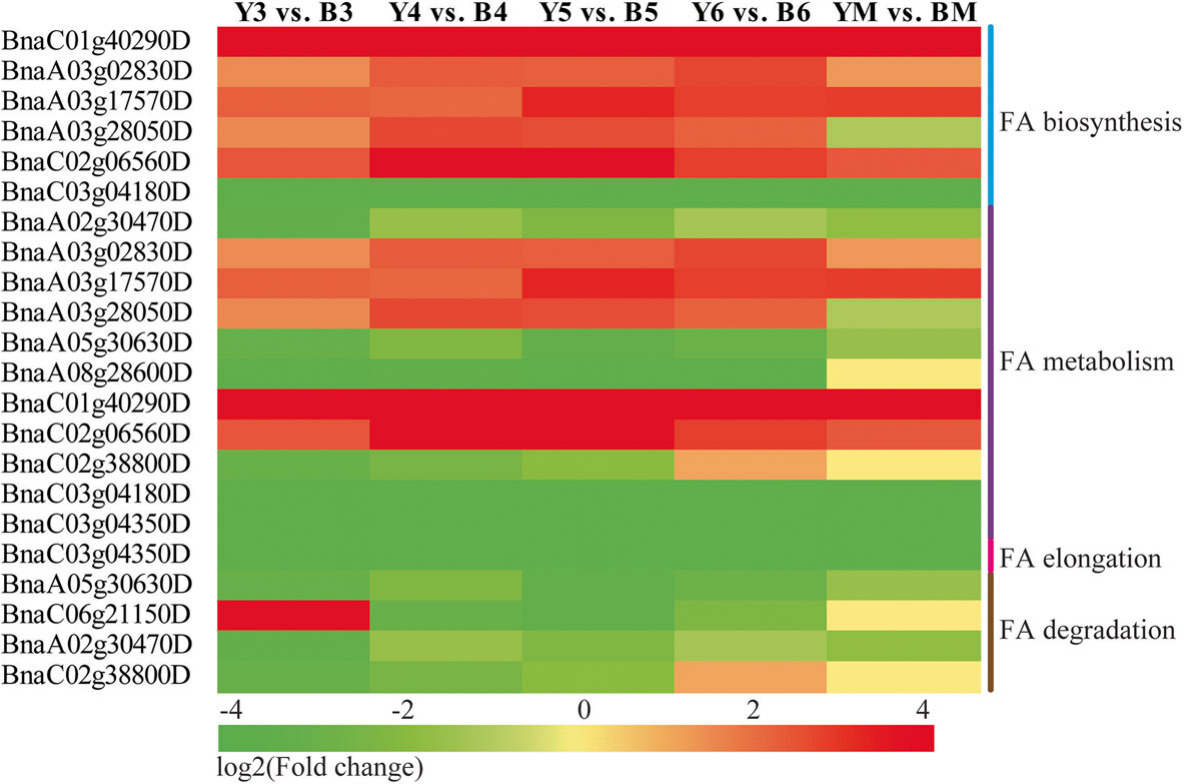
Heatmap of DEGs related to fatty acid biosynthesis and metabolism

We found that genes involved in FA biosynthesis, metabolism, and degradation were also changed (Fig. 5). For instance, five up-DEGs in yellow seed were involved in FA biosynthesis, encoding an NAD(P)-binding Rossmann-fold superfamily protein (BnaC01g40290D), a thioesterase superfamily protein (BnaA03g02830D), acetyl Coenzyme A carboxylase carboxyltransferase (BnaA03g17570D), stearoyl-acyl-carrier-protein desaturase (BnaA03g28050D), and chloroplastic acetylcoenzyme A carboxylase 1 (BnaC02g06560D). However, another homolog of the thioesterase superfamily protein (BnaC03g04180D) was downregulated in yellow seed compared with black seed. Five up-DEGs (BnaA03g02830D, BnaA03g17570D, BnaA03g28050D, BnaC01g40290D, and BnaC02g06560D) and six down-DEGs (BnaA02g30470D, BnaA05g30630D, BnaA08g28600D, BnaC02g38800D, BnaC03g04180D, and BnaC03g04350D) were involved in FA metabolism. BnaC03g04350D was also related to FA elongation. In addition, four down-DEGs (BnaC06g21150D, BnaA02g30470D, BnaA05g30630D, and BnaC02g38800D) were related to FA degradation. These DEGs might be related to the different oil content between yellow- and black-seeded *B. napus*.

### Real-time qPCR validates gene expression profiles

**Fig. 6:**
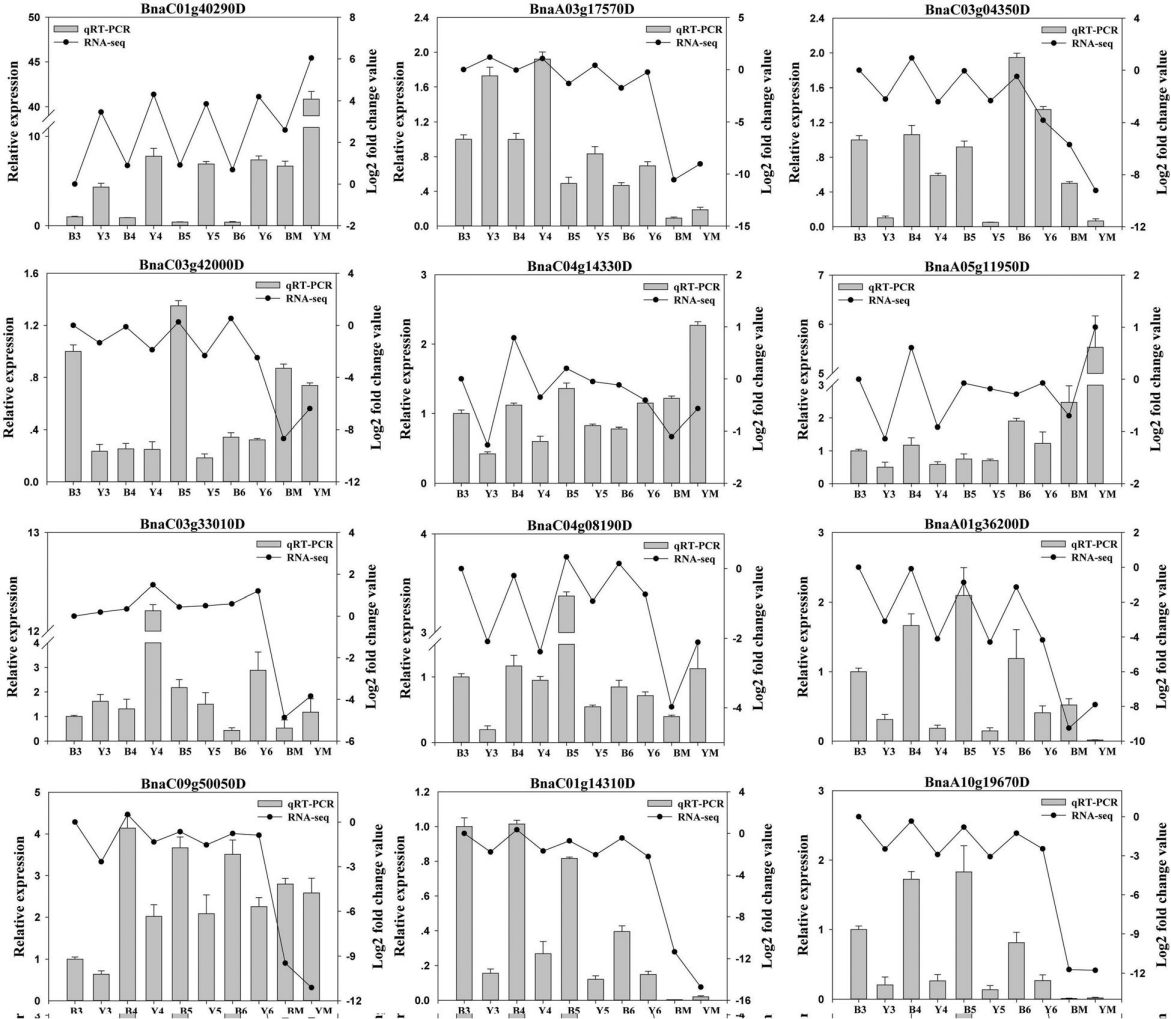
qPCR confirmation of DEGs between yellow- and black-seeded *B. napus*

To verify the sequencing results, DEGs involved in flavonoid and lignin biosynthesis, FA biosynthesis and metabolism were randomly selected for qPCR detection, including BnaC01g40290D encoding NAD(P)-binding Rossmann-fold superfamily protein/short-chain dehydrogenase/reductase (SDR), BnaA03g17570D encoding acetyl CoA carboxylase carboxyltransferase alpha subunit (CAC3), BnaC03g04350D encoding 3-hydroxyacyl-CoA dehydratase PASTICCINO 2 (PAS2), 4CL (BnaC03g42000D), C4H (BnaC04g14330D, BnaA05g11950D), LBD41 (BnaC03g33010D), PAL (BnaC04g08190D), BAN (BnaA01g36200D), CHI (BnaC09g50050D), LDOX (BnaC01g14310D) and CHS (BnaA10g19670D). The results showed that the qPCR data of the DEGs were in accordance with the sequencing results throughout seed development of yellow- and black-seeded *B. napus* (Fig. 6).

## Discussion

Due to the important economic value of rapeseed worldwide, research on improving seed quality has been carried out over the past half century, including reducing the erucic acid and glucosinolate contents and increasing the oleic acid content [43, 44]. However, breeding of rapeseed has been greatly hindered by the narrow genetic background either due to the short history or the artificial selection [31]. Previously, Wang et al. created yellow-seeded *B. napus* from the backcrossing progenies of *B. napus*-*S. alba* hybrids, including *S. alba* specific fragments in the 38 chromosomes [8, 34, 45]. RNA-Seq is a reliable method in analyzing the gene expressional level on the whole transcriptome level, and it also helps to reveal the expressional differences among different samples, which could somehow explain the different characteristics among samples. In the past decade, RNA-Seq has been broadly applied in expressional comparison among plant samples, and in finding alternative splicing, novel transcripts, SNP and InDels [46–48]. To identify the gene expression differences related to yellow seed character and accompanying quality variations, we carried out comparative transcriptome analysis between different developmental stages of yellow- and black-seeded *B. napus*. As reported by Qu et al. (2013), accumulation of polymeric phenolic compounds were similar in yellow and black rapeseeds, but the flavonoids were more accumulated in black seed since 3 WAF [49]. Thus, we collected the seeds at 3 WAF, 4 WAF, 5 WAF, 6 WAF and mature stage for RNA-Seq analysis. We found a total of 39,632 SNPs in ten libraries, including 1142 SNPs common in five developmental stages of black seed and 1543 SNPs common in five developmental stages of yellow seed, indicating that genomic differences exist between yellow- and black-seeded *B. napus*. We found a total of 15,251 DEGs among all the libraries, including 80 upregulated and 151 downregulated genes identified throughout yellow and black seed development, which might be related to seed development and FA accumulation (Additional file 2: Table S2). Regarding the DEGs between yellow and black rapeseed, we found that most were annotated to biosynthetic, pigment metabolic, and oxidation-reduction processes, such as flavonoid and phenylpropanoid biosynthesis, phenylalanine metabolism, flavone and flavonol biosynthesis, and FA biosynthesis and metabolism (Additional file 8: Figure S3, Additional file 9: Table S6). This agreed with Hong et al. who found that the downregulated DEGs in yellow seed coats were enriched in phenylpropanoid and flavonoid biosynthesis using yellow- and brown-seeded near-isogenic lines (NILs) of rapeseed as research materials [50]. Similar expression changes in *TT* genes have been reported in *B. juncea* seed coat [51].

Flavonoid biosynthesis has been considered to be the main pathway related to plant pigmentation, and *TT* genes related to it have been clarified in *A. thaliana*. Recently, homologs of *TTs* in *Brassica* have been comprehensively identified by Qu et al. [3], as well as the expression patterns in different yellow- and black-seeded *B. napus* inbred lines. In the present study, we compared the expression pattern of genes related to flavonoid biosynthesis in developing seeds of *B. napus* cv. ‘Yangyou 6’ and yellow-seeded *B. napus* selected from somatic hybrids of *B. napus*-*S. alba* (Fig. 4). Four homologs of *BnBAN* (BnaA01g36200D, BnaA03g60670D, BnaC01g29820D and BnaC04g18950D) were downregulated at two to four consecutive developmental stages of yellow rapeseed compared with black rapeseed. Two homologs of *BnDFR* (BnaA09g15710D and BnaC09g17150D) were also expressed at lower levels in yellow seed, which encodes an enzyme catalyzing dihydroflavanones to leucoanthocyanidins that was then converted to anthocyanidin [52]. *BnLDOX* (BnaC01g14310D, BnaA03g45610D and BnaC07g37670D) was downregulated in yellow seed, which encodes leucoanthocyanidin reductase and catalyzes the formation of anthocyanidins. Genes encoding chalcone synthase (BnaA10g19670D, BnaC09g43250D, BnaC03g06120D, BnaC02g05070D and BnaC08g26020D) were also downregulated throughout yellow seed development, which catalyzes the first step of flavonoid biosynthesis. *BnTT5* (BnaC08g26020D) was also downregulated in yellow seed compared with black seed at specific stages, encoding chalcone isomerase that is redundant and responsive to lights in Brassicaceae [53]. *BnTT8* (BnaC09g24870D and BnaA09g22810D) was also downregulated in yellow seed compared with black seed at specific stages, encoding the transcription factor bHLH as an important regulator throughout flavonoid biosynthesis. Another two *bHLHs* (BnaA01g17420D and BnaA01g24020D) were also downregulated, which have been proven to have functions in regulating jasmonate responses [54] and glucose homeostasis, and may affect ABA or salinity response in *Arabidopsis* [55]. In the present study, 21 *CYPs* were differentially expressed between yellow and black rapeseeds, although they were not shown to have functions in the secondary metabolism of *B. napus*. Previously, CYP71/75/93/81 have been reported to have functions in the biosynthesis of anthocyanin pigments and condensed tannins [42]. Lam et al. found that CYP75B4 and CYP93G1 promote tricin accumulation in *Arabidopsis* and *O. sativa*, which functions in generating 3′-hydroxylated flavonoids and flavone aglycones [56, 57]. Recently, Lei et al. found that a group of *CYPs*, including members of *CYP71/72/77/78/81/85/86/90/93*, were differentially expressed in *Dendrobium catenatum* from different locations and with different outlooks, which might be related to flavonoid biosynthesis [58]. *CYP86* has been reported to be involved in suberin monomer biosynthesis in *Arabidopsis* [59]. Expressional differences of five *TCPs* were also identified, which might be related to flavonoid biosynthesis since TCP3 could interact with R2R3-MYB [41]. In addition, *TCP1* directly activates *DWARF4* and promotes brassinosteroid biosynthesis [60].

**Fig. 7:**
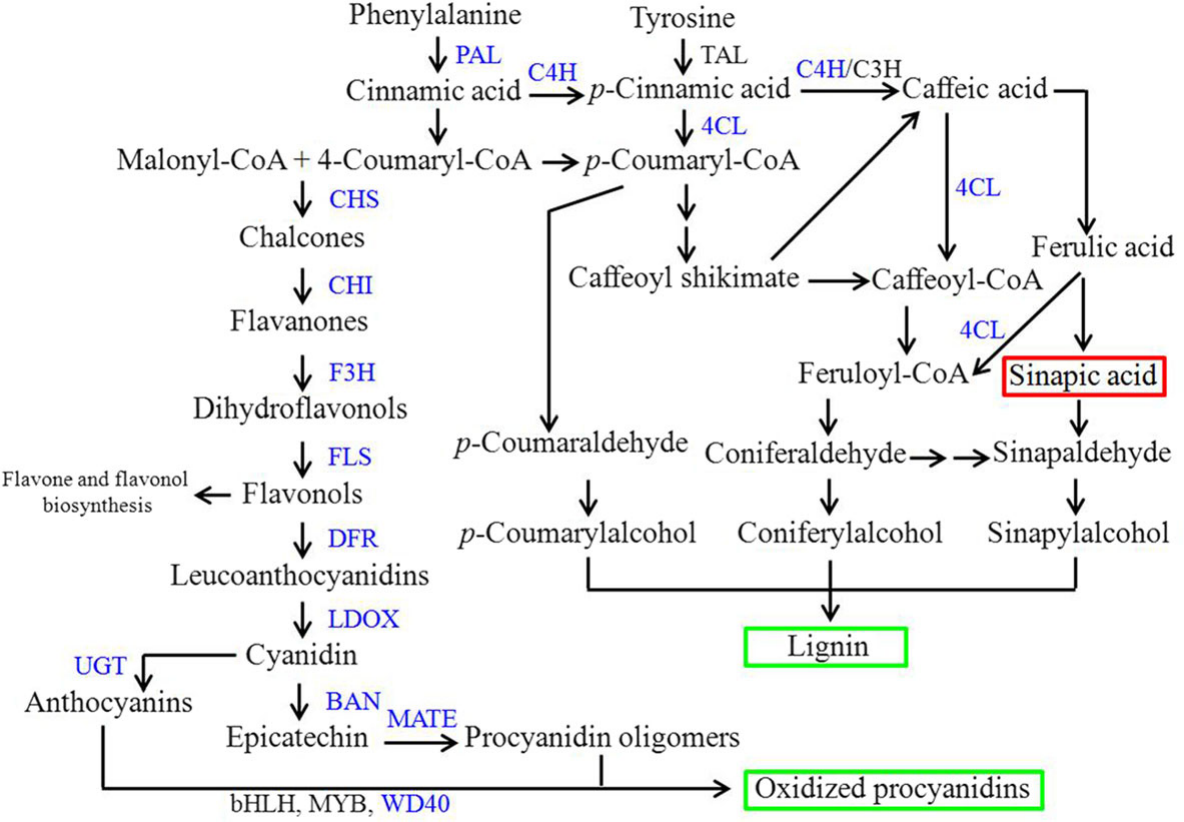
Proposed model for DEGs related to yellow seed quality. Blue font, downregulated genes in yellow seed compared with black seed. Red frame, increased chemical in yellow seed. Green frame, decreased chemical in yellow seed

Asides from flavonoids, lignin has been revealed to have a positive correlation with seed coat color [26]. We found that *4CL*, *C4H* and *PAL* were downregulated in yellow rapeseed compared with black seed, which play important roles in the less lignin in yellow seed (Fig. 4). PAL is involved in the first step of the phenylalanine metabolic pathway by catalyzing phenylalanine into cinnamic acid. Cinnamic acid was then transformed into coumaric acid by C4H. 4CL plays an important role in several steps of lignin biosynthesis and participates in the formation of coumaroyl-CoA, caffeoyl-CoA and feruloyl-CoA. Previously, Jiang et al. (2013) and Wang et al. (2018) reported that sinapic acid was more accumulated in yellow seed than black seed [39, 61]. Down-regulation of 4CL in yellow rapeseed indicated that caffeic acid and ferulic acid might be less transformed into caffeoyl-CoA and feruloyl-CoA. Thus, sinapis acid as another downstream chemical of caffeic acid and ferulic acid was more accumulated (Fig. 7). All these compounds are necessary intermediates of lignin biosynthesis [5]. Hong et al. found that the expression of genes involved in lignin biosynthesis were slightly but not markedly changed in yellow-seeded NILs [50]. As to the other DEGs related to secondary metabolism, we found genes encoding S-adenosylmethionine synthetase 2 (BnaA09g00390D), peroxidase (BnaA02g14050D, BnaC06g32660D, BnaC06g21080D), branched-chain amino acid transaminase 1 (BnaA01g36200D), methionine synthase 2 (BnaC03g33530D), aspartate aminotransferase 5 (BnaC01g06460D), phosphoglycerate kinase (BnaA07g34980D, BnaC02g46710D), lactate/malate dehydrogenase (BnaA09g16400D), glucose-1-phosphate adenylyltransferase family protein (BnaC04g33380D), beta glucosidase 19/28 (BnaA05g04030D, BnaC01g43700D, BnaC05g11190D, BnaA06g09700D), and pyruvate kinase (BnaA03g36910D) were downregulated in yellow rapeseed (Additional file 11: Figure S5). Peroxidase is one of three major enzymes involved in flavonoid oxidation through the associated reduction of hydrogen peroxide in the peroxidative cycle [62]. Hong et al. assigned peroxidase superfamily protein (e.g., peroxidase 4/7/25/52/53) to phenylpropanoid and flavonoid pathways, which were differentially expressed between yellow and black rapeseed NILs [50]. The DEGs related to flavonoid and lignin biosynthesis confirmed that they contribute to the different testa color.

Since the oil content of yellow seed was higher than that of black seed, we found 12 DEGs related to FA biosynthesis, metabolism, elongation and degradation (Fig. 5). In addition, we found that BnaA09g51510D (encoding pyruvate dehydrogenase E1 alpha) was upregulated throughout yellow seed development compared with black seed (Additional file 11: Figure S5). Its homolog in *A. thaliana* is important for seed oil biosynthesis. Plastidic pyruvate kinase (PKP) provides most of the pyruvate for plastidic FA synthesis, and the mutation of *PKP* severely impairs seed storage lipid synthesis [63]. BnaA03g17570D (acetyl Coenzyme A carboxylase carboxyltransferase alpha subunit) was upregulated in yellow seed. Its homolog in *A. thaliana* has been confirmed to be a subunit of heteromeric acetyl-CoA carboxylase (ACCase), which catalyzes the carboxyltransferase (CT) reaction. ACCase is responsible for the first step of FA synthesis [64]. Additionally, homolog of *UDP-glucose pyrophosphorylase* (*UGP*) (BnaA05g32480D), a sucrose-regulated protein, was upregulated in yellow seed, which is required for fumonisin B1-induced cell death [65]. A homolog of *beta glucosidase 25* (BnaC01g40610D) was also upregulated in yellow seed, which is involved in the carbohydrate metabolic process [66]. Upregulation of *cytochrome B5 reductase 1* (*CB5R1*) (BnaC02g02110D) in yellow seed might also be related to the FA variation, since CB5R is a microsomal membrane-bound protein that functions as part of the microsomal electron transfer system in FA desaturation [67]. CB5R can interact with ankyrin repeat-containing protein 2A (AKR2A), which interacts with ascorbate peroxidase 3 (APX3). APX3 can target peroxisomes [68]. This might be related to the above-mentioned expressional changes in genes encoding peroxidases. The expression changes of these genes may be helpful in explaining the FA variation in yellow seed and its adaptability to environments after flavonoid reduction.

## Conclusions

In the present study, whole transcriptome gene expression was analyzed in developing seeds of yellow-seeded *B. napus* derived from hybrids of *B. napus-S. alba*, and black-seeded *B. napus*. We identified the DEGs with seed development, which might be related to the development and biosynthetic process. In addition, DEGs related to the quality difference between yellow and black rapeseed have been identified, which mainly participate in flavonoid biosynthesis, phenylpropanoid biosynthesis, phenylalanine metabolism, flavone and flavonol biosynthesis, fatty acid biosynthesis and metabolism. These down-regulated genes are helpful to explain the less pigmentation (e.g. *CHS*, *CHI*, *F3H*, *FLS*, *DFR*, *LDOC*, *BAN*) and lignin (*PAL*, *C4H* and *4CL*), and higher oil content in yellow rapeseed compared with black seed (Fig. 7). Future functional analysis of these genes would contribute to the molecular dissection of yellow seed character in *B. napus*.

## Methods

### Plant material

Yellow-seeded *B. napus* (line W82) was preserved in our lab and was selected from back-crossing progenies of somatic hybrids of *B. napus*-*S. alba*. The black-seeded rapeseed (*B. napus* cv. ‘Yangyou 6’) was obtained from the Jiangsu Lixiahe Region Agricultural Research Institute, China [45]. Both rapeseed lines were grown in the experimental field of Yangzhou University, Yangzhou, China. In addition to the visible difference in seed coat color, the flavonoid content in yellow seed was lower than that in black seed, and the seed FA composition and content were different between yellow- and black-seeded *B. napus*. The oil content of W82 was 6% higher than that of Yangyou 6. Higher protein and sucrose contents, less dietary fiber and crude fiber, and fewer glucosinolates accumulated in the seed meal of yellow rapeseed compared with black rapeseed (Additional file 12: Table S7) [8, 39, 61, 69]. The developing seeds at 3 WAF, 4 WAF, 5 WAF, 6 WAF, and mature seeds were collected from three pods each of ten individual plants for comparative analysis. During the seed development, differentially accumulated pigments were visual since 5 WAF of yellow and black rapeseeds. Proanthocyanidins (PAs) were less accumulated throughout yellow seed development than black seed [39, 70].

### RNA extraction, library construction, and RNA sequencing

For each developmental stage of each rapeseed line, five RNA samples were separately isolated from polled seeds of ten plants using Trizol Reagent Solution (Invitrogen, USA). Each library was pooled by mixing equal quantities of five RNA samples. The RNA quality was validated using agarose gel electrophoresis, Nanodrop, Qubit, and Agilent 2100 to confirm the purity, concentration, and integrity, respectively. mRNA was purified using beads with Oligo (dT), and cDNA was then synthesized with random hexamers after fragmentation of mRNA. After purification of cDNA with AMPure XP beads, unique adaptors and indexes were ligated. Certain fragments were selected with beads and enriched using PCR amplification. Finally, ten cDNA libraries for five developmental stages (3 WAF, 4 WAF, 5 WAF, 6 WAF and mature seeds) of yellow- and black-seeded *B. napus* were normalized based on a Qubit assessment, and the insert size was validated by Agilent 2100. Then, the polled libraries were sequenced by the Illumina HiSeq™ 2000 platform.

### Bioinformatics analysis

After removing adaptor sequences and low-quality sequences, clean reads were mapped to the *B. napus* cv. Darmor-*bzh* genome (version 5) using TopHat2 [71, 72]. Based on the predicted gene models of Cufflinks, classification and statistics of alternative splicing were carried out using AS profile [73, 74]. Novel transcripts were predicted using Cufflinks to assemble the mapped reads on the genome and compare it with the known gene models [75]. The abundance of reads mapped to reference was estimated and normalized using Reads Per Kilo bases per Million reads (RPKM), and all the transcripts with RPKM value> 1 were used for further analysis. Differential gene expression analysis was performed using DESeq [76, 77], and DEGs between libraries were screened with a threshold of | (FoldChange)| > 1 and q value< 0.005. All the DEGs among different libraries were clustered base on the log_10_(RPKM+ 1) and log_2_(Fold Change) value. Gene ontology (GO) enrichment of the DEGs was performed using GOseq with a corrected *p*-value < 0.05 [78], and KEGG pathway enrichment of DEGs was performed using KOBAS 2.0 with a corrected *p*-value < 0.05 [79]. An overview of pathways related to these DEGs was predicted by MapMan (version 3.6.0) analysis [80].

### cDNA synthesis and qRT-PCR validation

Subsamples of RNA-Seq were reverse transcribed into cDNA for real-time qPCR validation using the Revert Aid First Strand cDNA Synthesis Kit and SYBR Green Real-Time PCR Master Mixes (Thermo, USA). qRT-PCR was performed on a fluorescence quantitative system Mx3005P (Agilent, USA). Genes, primers and size of the amplicon are listed in Additional file 13: Table S8. *B. napus β-actin* (NCBI AF111812) was used as an endogenous control to generate the △Ct for three technological replicates.

## Additional files

Additional file 1: **Table S1.** Summary of read mapping in RNA-Seq analysis of yellow and black rapeseeds. B3, B4, B5, B6 and BM indicate 3 ~ 6 WAF and mature black seed. Y3, Y4, Y5, Y6 and YM indicate 3 ~ 6 WAF and mature yellow seed. (XLSX 34577 kb)

Additional file 2: **Table S2.** DEGs identified with black or yellow seed development and between same developmental stages of two rapeseeds. (XLSX 11030 kb)

Additional file 3: **Table S3.** DEGs related to seed development. (XLSX 94 kb)

Additional file 4: **Table S4.** Up- and downregulated genes among all the developmental stages of yellow- and black-seeded *B. napus*. (XLSX 25 kb)

Additional file 5 **Figure S1.** The SOM clusters of DEGs from five developmental stages of two rapeseed lines. (XLSX 173 kb)

Additional file 6: **Table S5.** All the up- and down-DEGs with GO annotation. (XLSX 63 kb)

Additional file 7: **Figure S2.** KEGG enrichment of DEGs (Y3 vs. B3) in flavonoid and phenylpropanoid biosynthesis. (XLSX 24 kb)

Additional file 8: **Figure S3.** KEGG enrichment of up- and down-DEGs between yellow- and black-seeded *B. napus*. (XLS 31 kb)

Additional file 9: **Table S6.** KEGG pathways of DEGs between yellow- and black-seeded *B. napus*. (PDF 519 kb)

Additional file 10: **Figure S4.** Overview of pathways related to the DEGs between yellow- and black-seeded *B. napus*. (PDF 981 kb)

Additional file 11: **Figur S5.** Heatmap of DEGs involved in secondary metabolism. (JPG 6164 kb)

Additional file 12: **Table S7.** Quality differences between yellow- and black-seeded *B. napus. (JPG 1896 kb)*

Additional file 13: **Table S8.** Primers for qPCR validation of DEGs (JPG 3234 kb)

## Abbreviations

4CL: 4-coumarate CoA ligase
ACCase: Acetyl-CoA carboxylase
AKR2A: Ankyrin repeat-containing protein 2A
APX3: Ascorbate peroxidase
C4H: Cinnamate 4-hydroxylase
CB5R1: Cytochrome B5 reductase 1
CT: Carboxyltransferase
CYP: Cytochrome P450
DEG: Differentially expressed gene
EBG: Early biosynthesis gene
FA: Fatty acid
GO: Gene ontology
GWAS: Genome wide association study
KEGG: Kyoto encyclopedia of genes and genomes
LBG: Late biosynthesis gene
LEA: Late embryogenesis abundant hydroxyproline
MBW: MYB-bHLH-WD40
NIL: Near-isogenic line
PA: Proanthocyanin
PAL: Phenylalanine ammonia-lyase
PKP: Pyruvate kinase
qRT-PCR: Quantitative real-time polymerase chain reaction
RPKM: Reads per kilo bases per million reads
SNP: Single nucleotide polymorphism
TT: Transparent testa
UGP: UDP-Glucose pyrophosphorylase
WAF: Weeks after flowering
