# Supplementary material for: Transcriptomic comparison between developing seeds of yellow- and black-seeded Brassica napus reveals that genes influence seed quality
Source: BMC Plant Biol. 2019 May 16;19:203. doi: 10.1186/s12870-019-1821-z (PMC6524335; doi:10.1186/s12870-019-1821-z)
Supplement: Supplementary file 9 — Table S6. KEGG pathways of DEGs between yellow- and black-seeded B. napus. (PDF 519 kb) [file 12870_2019_1821_MOESM9_ESM.pdf]

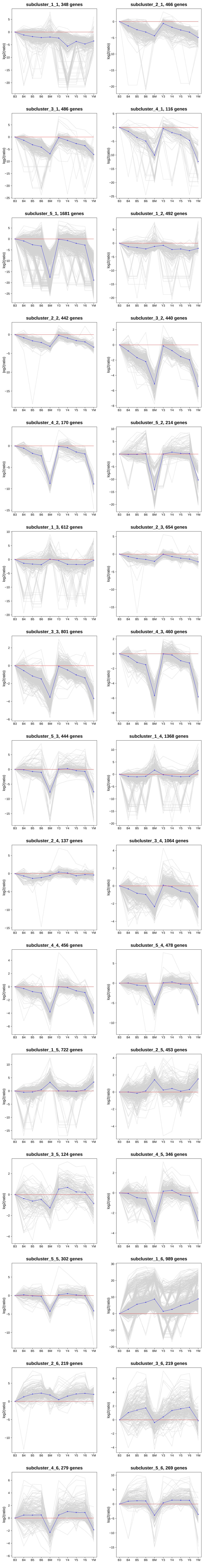
subcluster_1_1, 348 genes
subcluster_2_1, 466 genes
subcluster_3_1, 486 genes
subcluster_4_1, 116 genes
subcluster_5_1, 1681 genes
subcluster_1_2, 492 genes
subcluster_2_2, 442 genes
subcluster_3_2, 440 genes
subcluster_4_2, 170 genes
subcluster_5_2, 214 genes
subcluster_1_3, 612 genes
subcluster_2_3, 654 genes
subcluster_3_3, 801 genes
subcluster_4_3, 460 genes
subcluster_5_3, 444 genes
subcluster_1_4, 1368 genes
subcluster_2_4, 137 genes
subcluster_3_4, 1064 genes
subcluster_4_4, 456 genes
subcluster_5_4, 478 genes
subcluster_1_5, 722 genes
subcluster_2_5, 453 genes
subcluster_3_5, 124 genes
subcluster_4_5, 346 genes
subcluster_5_5, 302 genes
subcluster_1_6, 989 genes
subcluster_2_6, 219 genes
subcluster_3_6, 219 genes
subcluster_4_6, 279 genes
subcluster_5_6, 269 genes
log2(ratio)
B3 B4 B5 B6 BM Y3 Y4 Y5 Y6 YM
