# Supplementary figures and images for: Transcriptomic comparison between developing seeds of yellow- and black-seeded Brassica napus reveals that genes influence seed quality
Source: BMC Plant Biol. 2019 May 16;19:203. doi: 10.1186/s12870-019-1821-z (PMC6524335; doi:10.1186/s12870-019-1821-z)

## FLAVONOID BIOSYNTHESIS

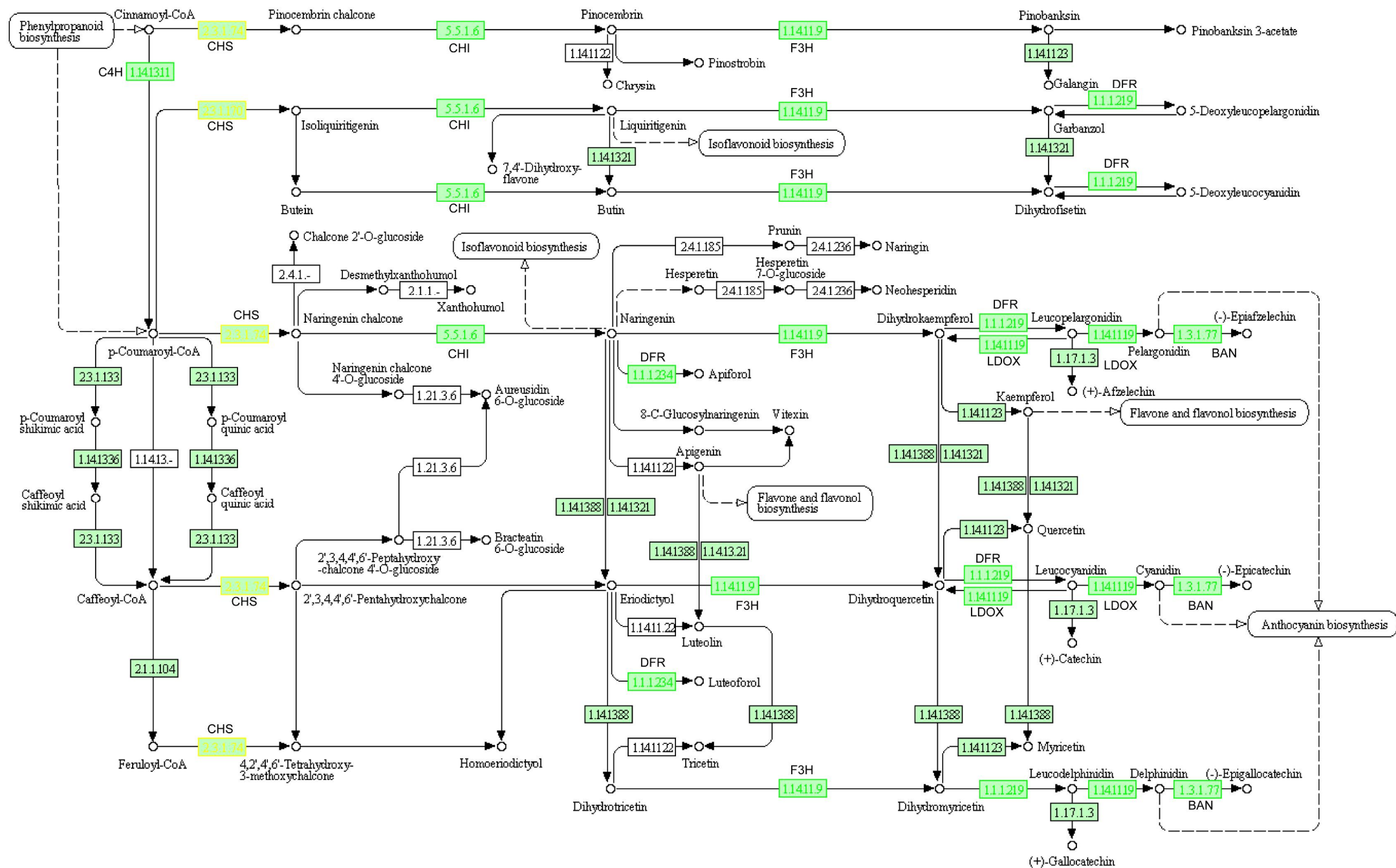

## PHENYLPROPANOID BIOSYNTHESIS

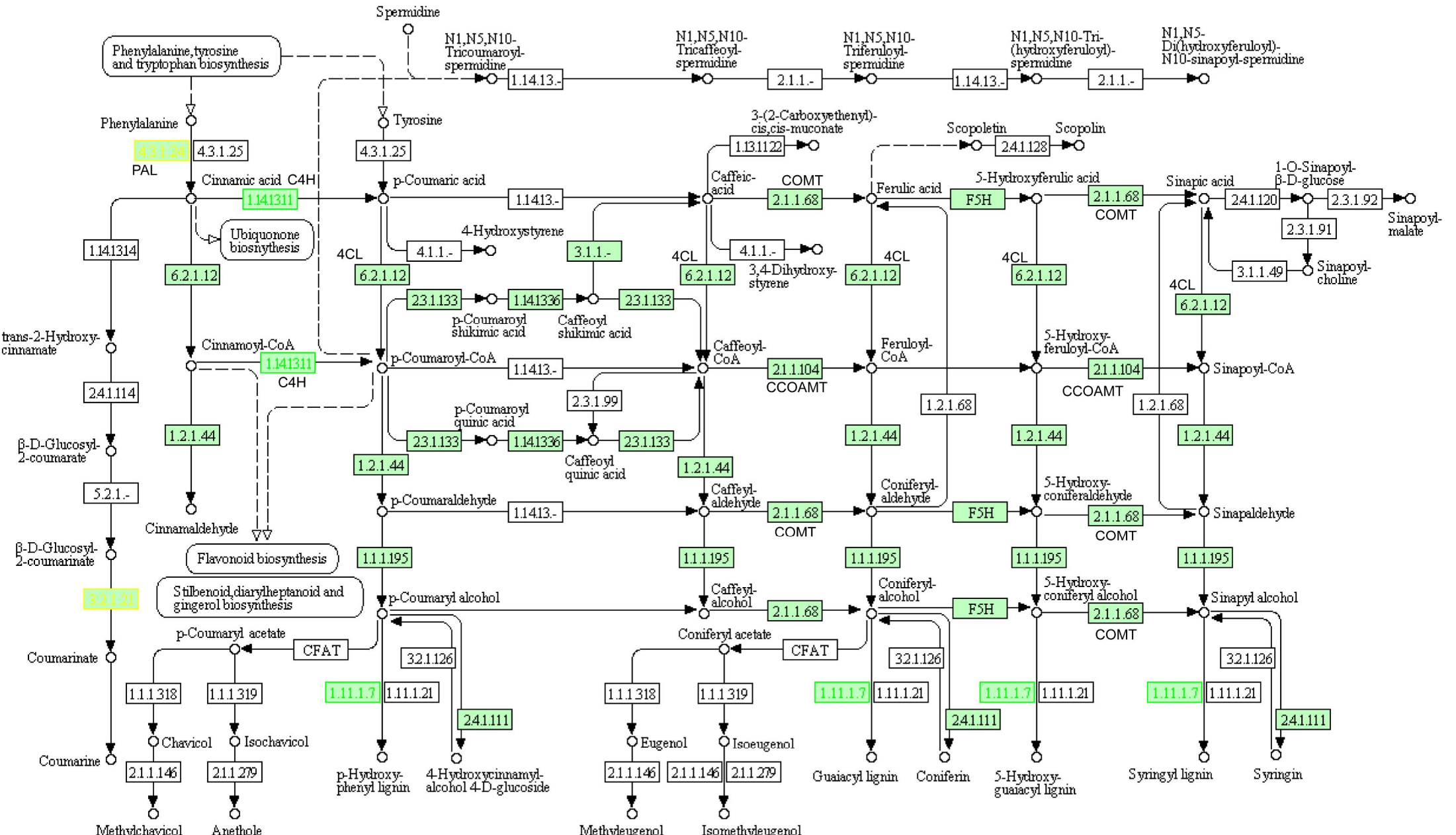

Supplement: Supplementary file 10 — Figure S4. Overview of pathways related to the DEGs between yellow- and black-seeded B. napus. (PDF 981 kb) [file 12870_2019_1821_MOESM10_ESM.pdf]

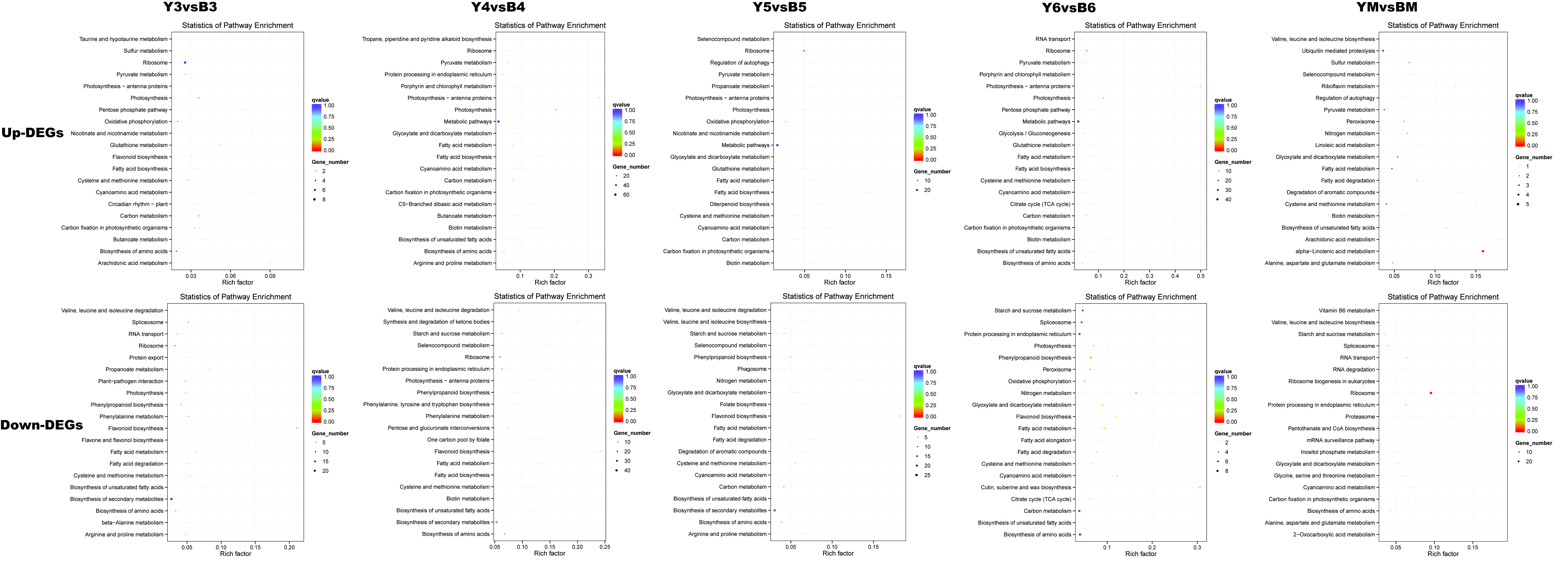

Supplement: Supplementary file 11 — Figur S5. Heatmap of DEGs involved in secondary metabolism. (JPG 6164 kb) [file 12870_2019_1821_MOESM11_ESM.jpg]

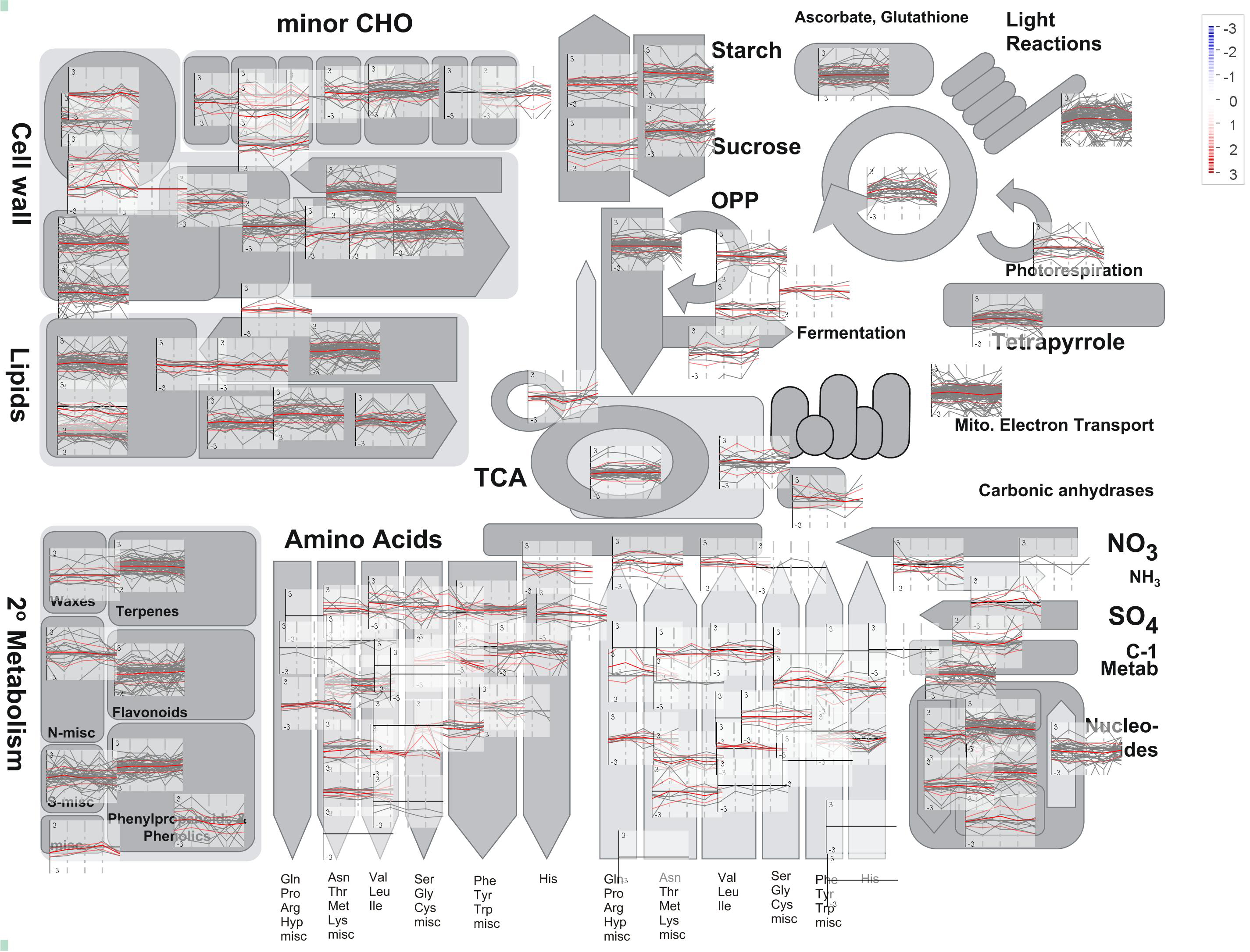

Supplement: Supplementary file 12 — Table S7. Quality differences between yellow- and black-seeded B. napus. (JPG 1896 kb) [file 12870_2019_1821_MOESM12_ESM.jpg]

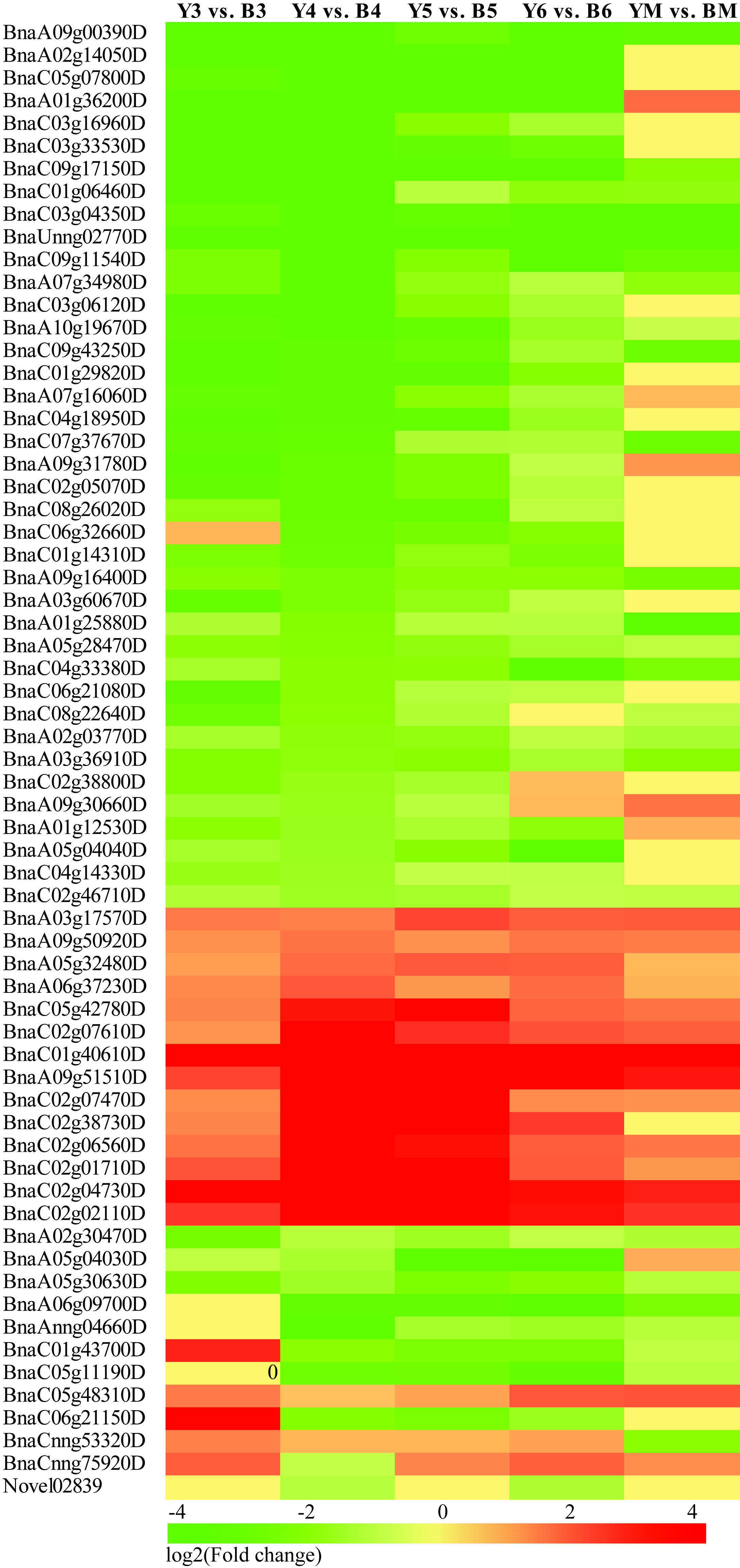

Supplement: Supplementary file 13 — Table S8. Primers for qPCR validation of DEGs (JPG 3234 kb) [file 12870_2019_1821_MOESM13_ESM.jpg]
